# Supplementary material for: Fruiting Body Heterogeneity, Dimorphism and Haustorium-like Structure of Naematelia aurantialba (Jin Er Mushroom)
Source: J Fungi (Basel). 2024 Aug 7;10(8):557. doi: 10.3390/jof10080557 (PMC11355420; doi:10.3390/jof10080557)
Supplement: Supplementary file 1 [file jof-10-00557-s001.zip › jof-3092700-supplementary.pdf]

Supplementary materials

Fruiting Body Heterogeneity, Dimorphism and Haustorium-Like Structure of *Naematelia aurantialba* (Jin Er Mushroom)

Ying Yang [1,2] and Caihong Dong [1,*]

[1] State Key Laboratory of Mycology, Institute of Microbiology, Chinese Academy of Sciences, Beijing 100101, China; yangy@im.ac.cn

[2] University of Chinese Academy of Sciences, Beijing 100049, China

* Correspondence: dongch@im.ac.cn; Tel./Fax: +86-010-64806138

Information

1. Supplementary Figures S1 to S2: page 2-3

Figure S1.

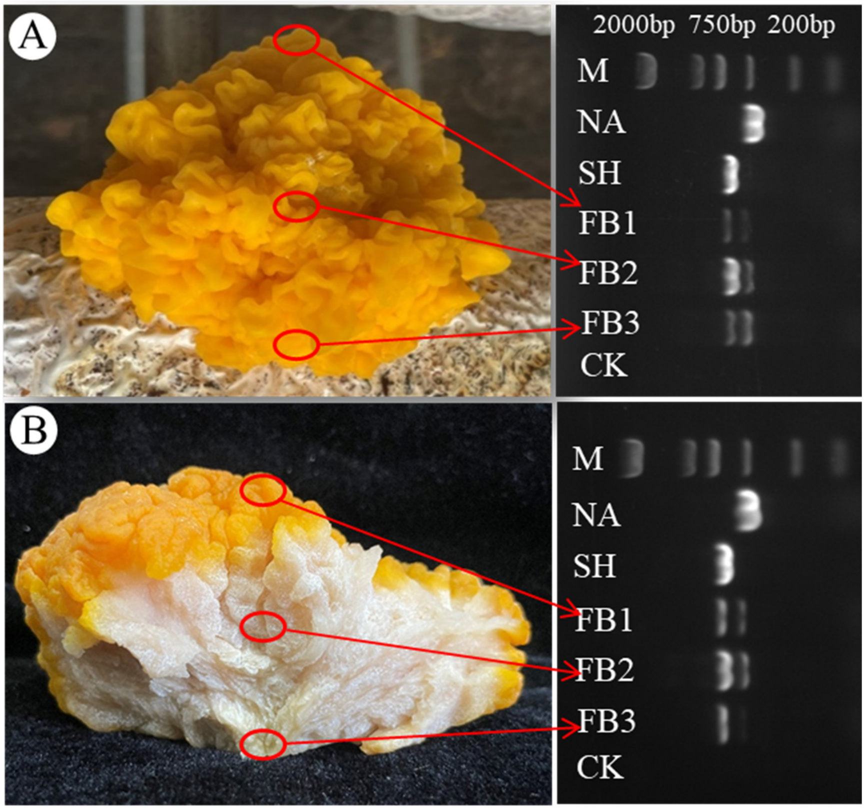

Figure S1. The fruiting body heterogeneity of Jin Er Mushroom.

(A, B) Artificially cultivated Jin Er. M: marker 2kb; NA: *N. aurantialba* basidiospores; SH: *S. hirsutum* pure mycelia; FB1 and FB2 and FB3: Top, mid and bottom of Jin Er fruiting body circled in red; CK: negative control.

Figure S2.

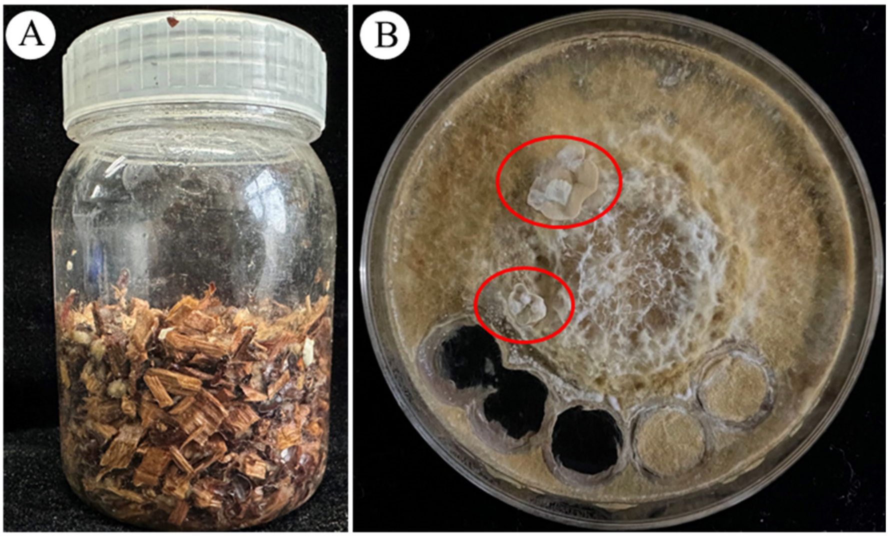

Naematelia aurantialba *Stereum hirsutum*

Figure S2. The fruiting body cultivation of pure spawn of *Naematelia aurantialba* and *Stereum hirsutum*.

(A) No mycelial growth and fruiting body development cultivated with pure spawn of *Naematelia aurantialba*; (B) The fruiting body cultivated with pure spawn of *Stereum hirsutum* (circled in red).
